# Efficacy of closed cell wet-suit at various depths and gas mixtures for thermoprotection during military training dives

**DOI:** 10.3389/fphys.2023.1165196

**Published:** 2023-05-24

**Authors:** Karen R. Kelly, Laura J. Palombo, Andrew E. Jensen, Jake R. Bernards

**Affiliations:** ^1^ Applied Translational Exercise and Metabolic Physiology Team, Warfighter Performance, Naval Health Research Center, San Diego, CA, United States; ^2^ Leidos, Inc., San Diego, CA, United States

**Keywords:** military, cold-water, skin temperature, core temperature, SCUBA

## Abstract

**Purpose:** To evaluate a closed-cell wet-suit for thermal protective capability during extreme cold water exposure at various depths.

**Methods:** Thirteen (n = 13) elite military divers who were tasked with cold-water training, participated in this study. To mimic various depths, the Ocean Simulation Facility (OSF) at the Navy Experimental Diving Unit (NEDU) was pressurized to simulate dive depths of 30, 50, and 75fsw. Water temperature remained at 1.8–2.0°C for all dives. Four divers dove each day and used the MK16 underwater breathing apparatus with gas mixes of either N202 (79:21) or HeO2 (88:12). Mean skin temperature (T_SK_) (Ramanathan, 1964), core temperature (Tc), hand and foot readings were obtained every 30 min for 30 and 50fsw and every 15 min during the 75fsw dive.

**Results:** T_C_ was significantly reduced across all dives (*p* = 0.004); however, was preserved above the threshold for hypothermia (post dive Tc = 36.5 ± 0.4). There was no effect of gas mix on T_C_. T_SK_ significantly decreased (*p* < 0.001) across all dives independent of depth and gas. Hand and foot temperatures resulted in the termination of three of the dives. There were no significant main effects for depth or gas, but there were significant main effects for time on hand temperature (*p* < 0.001) and foot temperature (*p* < 0.001).

**Conclusion:** Core temperature is maintained above threshold for hypothermia. Variatioins in T_C_ and T_SK_ are a function of dive duration independent of depth or gas for a closed-cell wet-suit in cold water at various depths. However, both hand and foot temperatures reached values at which dexterity is compromised.

## Introduction

Diving and operating in an undersea environment present many challenges to the human system. We recently showed that divers have an increased stress response elucidated from prolonged cold-water diving and there is an increase in metabolic demand to maintain core temperature (Chapin et al., 2021; Kelly et al., 2022). Further, immersion in water <15°C initiates an increase in sympathetic activity known as the “cold shock response,” which is intended for survival but in some conditions may come at the cost of loss of dexterity or injury to the extremities (M. Tipton, 1989; M. J; Tipton, 1989). Upon entry into cold water, skin cooling begins within 3 min, triggering activation of α-adrenergic receptors and elevation of norepinephrine concentrations, leading to veno- and vasoconstriction, and ultimately a decrease in peripheral blood flow (Sramek et al., 2000; M; Tipton, 1989; M. J; Tipton, 1989). Movement of blood to the core is exacerbated by the pressure of water submersion, which effectively shunts blood to the core via peripheral vasoconstriction (Stocks et al., 2004; M; Tiptdon, 1989). This shift of blood flow preserves core temperature and function of vital organs. However, with minimal heat input to the extremities, “physiological amputation” occurs increasing susceptibility of extremities to non-freezing cold injury (Havenith et al., 1995; Heus et al., 1995; Tipton et al., 2017), which poses a threat to individuals exposed to extreme cold.

Traditionally military divers don dry-suits in cold water to maintain core, skin, and extremity temperature, enabling diving in suboptimal temperatures. Dry-suits typically have multiple layering components, which make them impermeable to cold water, yet still risk thermal injury if the system is compromised. Neoprene wet-suits increase mobility and are flexible, and lighter in weight. Closed-cell neoprene is a synthetic rubber that has been foamed to create bubbles of nitrogen gas which helps preserve warmth. However, heat can still be lost through conduction, and at depths over 15 m, heat is lost at three times the rate at the surface due to compression of the material (Amini et al., 2013). Recently, we demonstrated these suits can preserve core temperature for long durations in extremely cold water at 20fsw)(Kelly et al., 2022). However, to expand the use case of these wet-suits to other occupations and across other dive profiles (e.g., pararescue jumpers, salvage divers, coast guard, oil rig workers, watercraft maintenance) there is need to evaluate whether these wet-suits can effectively thermoregulate at increased depth and with different gas mix.

There is scarce and conflicting literature on core temperature decrease, extremity temperature regulation and respiratory heat loss when breathing different gas mix during moderate depth dives (Hoar et al., 1976; Spitler et al., 1980; Mitchell & Doolette, 2013; Jones et al., 2023). What is known is that there is a difference in thermal conductivity of two gas mix, heliox (HeO_2_) and nitrox (N_2_O_2_), with HeO2 having an increased thermal conductivity than N_2_O_2_ which may reduce core temperature and lead to hypothermia (Mitchell & Doolette, 2013; NOAA, 2023; StatPearls, 2023). Moreover, respiratory heat loss is a known phenomenon when breathing cold air especially during exercise (Castellani et al., 2006; Mortola & Maskrey, 2011). However, to our knowledge there is no current data on heat loss [thermoregulation] wet-suit with different dive gas-mixes in divers donning neoprene [wet-suits]. Recent work in Canadian military divers suggest that divers were more comfortable in neoprene gloves and that hand remained warmer than in dry-suits (Sullivan-Kwantes & Tikuisis, 2023) in arctic waters; however the duration of exposure was short, between 5–37 min. Thus, the purpose of this study was to expand on the body of knowledge with respect to closed cell neoprene [wet-suits] during extreme cold-water diving and evaluate thermal strain in military divers across three different depths and two typical gas mixes. The goal was to determine if closed cell wet-suits were effective at thermoprotection especially at the extremities where military divers are vulnerable to non-freezing cold injuries (NFCI). We hypothesize, based upon previous work that despite compression, closed cell wet-suits will effectively defend again hypothermia and maintain extremity temperature above the threshold for NFCI independent of gas mix.

## Material and methods

Participants. Thirteen (n = 13), active-duty elite military divers that were tasked with cold-water training participated in this study. This study was approved by the Institutional Review Board at Naval Health Research Center and adhered to Department of the Navy human research protection policies (Protocol NHRC. 2017.0019). All participants provided written informed consent.

### Dive procedure

**FIGURE 1:**
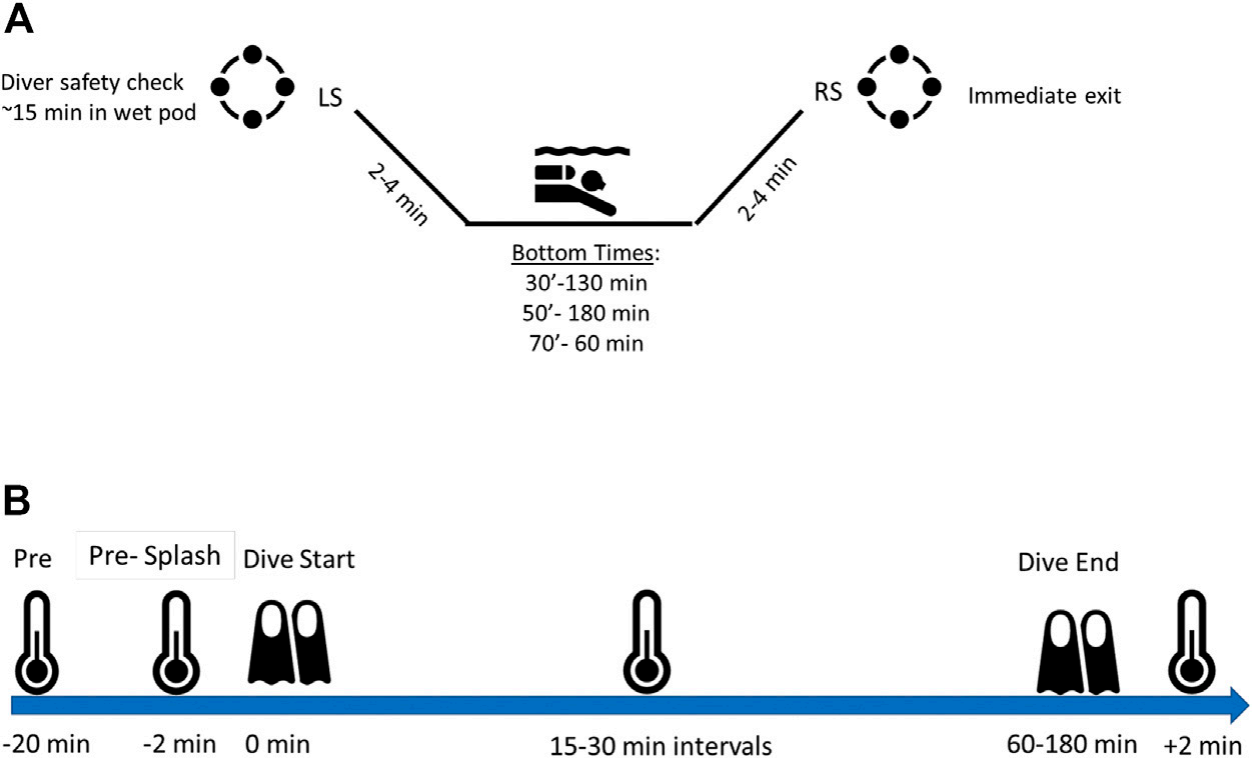
Schematic of dive times **(A)** and thermal readings **(B)** LS- leave surface, RS-return to surface.

To mimic cold-water at various depths, the Ocean Simulation Facility (OSF) at Navy Experimental Diving Unit (NEDU) was utilized across six diving days (one dive per day for six total dives). Water temperature was maintained 1.8°C–2.0°C (35.3°F–36.0°F). Dive depths were simulated using the OSF, which was pressurized to simulate depths of 30, 50, and 75 feet of sea water (fsw)(9.1, 15.2, and 22.9 m(m)). Dive time started when divers entered the water. Press time to depth took less than 3 min. Dive profiles were based upon military training dive profiles and varied based upon depth (Figure 1A). Bottom times were planned for 180, 180 and 60 min for 30, 50, and 75fsw (9.1, 15.2, and 22.9 m) respectively. Divers once at depth remained passive in a seated or semi-standing buoyant position. Divers moved minimally to port hole for temperature readings as indicated in Figure 1B. Movement from resting position to port hole was approximately 4–5 feet.

Divers donned wet-suits (Yazbeck, Redondo Beach, CA) that were made of closed cell 10-mm-thick neoprene were worn with a hood. The “Farmer John” configuration consisted of a two-piece garment which consisted of a long neoprene pants (to the ankles) with a sleeveless neoprene top. Over this, the second layer was a long-sleeve neoprene top (to the wrists) with a beaver tail, resulting in 20-mm (mm) of neoprene covering the torso. Divers wore two layers of 5-mm-thick gloves and booties, for 10 mm of protection on hands and feet. Divers used the MK16 underwater breathing apparatus (UBA) which is a closed-circuit rebreather. Inspired gas mixes of either N2O2 (79:21, N2:O2) or HeO2 (88:12, He:O2). These were training dives to evaluate the efficacy and expand the use case of this type of wet-suit under potential operational conditions^.^

### Thermoregulation

The night before data collection, single use ingestible core temperature capsules (Vital Sense, Philips Respironics, Bend, Oregon) were activated and distributed with instructions to ingest the capsule at 0500 on the day of the dive, which was approximately 4 h prior to dive time. Thermistor-based capsules have a sensing range of 25°C–50°C with reported accuracy ±0.1°C. Capsules pass through the GI tract without affecting bodily functions and are easily passed. Prior to dive and before wet-suits were donned, skin temperature patches (Vital Sense, Philips Respironics, Bend, Oregon) were affixed to the right side of the participant at the following sites: dorsal hand, dorsal foot, mid pectoralis (chest), arm (lateral deltoid (arm)), mid-thigh (thigh), and mid-calf (leg). Reported accuracy of the skin patches was ±0.25°C (−20°C–32°C). Mean skin temperature was calculated using four of the six sites; chest, arm, thigh, and leg (Ramanathan, 1964). Readings were collected every 15 minutes throughout each dive. Temperature values are reported from each hour of the dive. As a thermal safety precaution, dives were terminated if/when skin temperature at any location dropped below 10.0°C (50.0°F) or if/when core temperature dropped below 35.0°C (95.0°F). Dives were also terminated for any equipment malfunction or upon diver’s request. Prior to donning gloves and booties finger and toe temperatures were measured via infrared laser. Immediately post-dive within 1 min of exiting the water, gloves, and booties, were removed finger and toe temperatures were measured.

### Statistical analysis

As the purpose of this study was to determine the effectiveness of closed-cell neoprene suits during various diving conditions, multiple polynomial regression models were fit to each dive depth. Prior to any model development, a between-within repeated measures factorial ANOVA (split plot ANOVA) was run with depth as a between factor (3 levels) and gas (2 levels) and time (2 levels) as within factors (alpha level *p* < 0.05). It was determined that gas mixture did not influence temperature changes at any dive depth dive except for foot temperature. Therefore, models were built by combining datasets for gas at each dive depth. Models included site specific temperature readings as the outcome variable and dive time at the predictor to examine the rate of change in temperature at various body sites as a function of time. The adjusted R2 was used to determine the optimal ordered polynomial which best fits the given data set. All statistical models were completed using R version 4.2.1.

## Results

Participants. Participant’s demographics are in Table 1 All participants were experienced military divers and were tasked with cold-water dive training. Prior to this training, none had engaged in cold-water diving analogous to the conditions presented herein. Final dive times were 130, 180, and 60 min for 30 (9.1 m) 50 (15.2 m) and 75fsw (22.9 m) dives. The 30fsw was terminated early due to one diver’s foot reaching 10°C.

**TABLE 1:** Demographic data.

Measure	Baseline
**Age,** yrs	30 ± 6
**Height,** cm	182 ± 4
**Weight,** kg	86.8 ± 6.8
**BMI**	26.1 ± 0.9
**Body Surface Area,** m^2^	2.08 ± 0.03
n = 13. Data are presented as Mean ± Standard Deviation	

**FIGURE 2:**
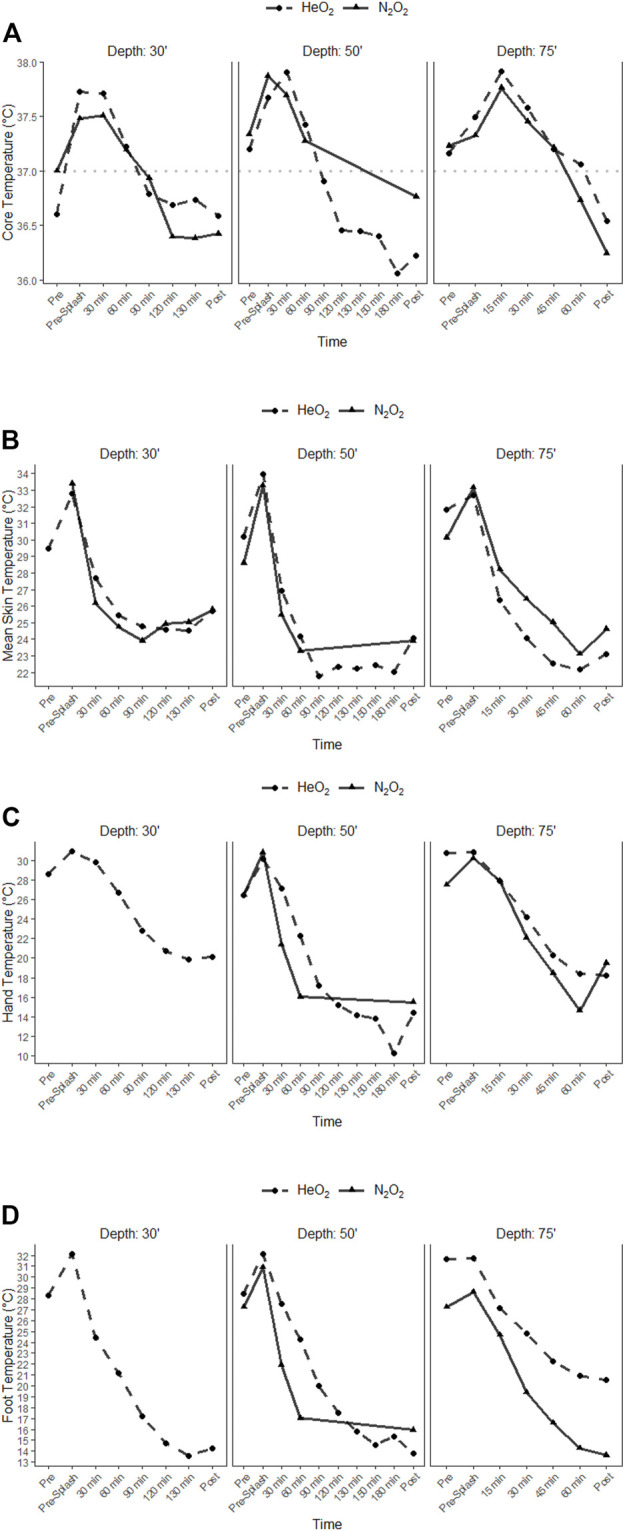
Temperature change between different dive depths and gas mix for core **(A)** mean skin **(B)**, hand **(C)** and foot **(D)**. Solid line indicates N2O2 and dashed line HeO2.

Core Temperature. There were no significant main effects for depth or gas, but there were significant main effects for time for core temperature, (*p* = 0.004, ηp2 = .77). Mean core temperatures are shown in Figure 2A; where solid lines indicate N_2_O_2_ gas mix and dashed lines indicate HeO_2_. The dashed line across the middle of the figure depicts a resting baseline core temperature of 37.0°C (98.6°F). Core temperature increased from baseline (prior to donning wet-suit) to pre-splash (wet-suit donned, “jock up phase”) participants independent of dive and participant due to the insulating properties of the wet-suit. Upon entry into the cold water, core temperature decreased as dive duration increased. No diver reached the safety threshold of core temperature below 35.0°C (95.0°F). The lowest individual core temperature recorded was 35.8°C (96.4°F); which occurred following the 75fsw (22.9 m) dive using N_2_O_2_. The rate of core temperature change across dive depth is a function of time in the water and independent of gas mix. The respective adjusted R2 indicates that time accounts for 52%–82% of the variance. When analysed by each gas for each dive depth (Figure 2B), the adjusted R2 values indicate that time accounts for 52%–71% of the variance when breathing N_2_O_2_ and 45%–81% of the variance when breathing HeO2.

Mean Skin Temperature. As observed with core temperature a significant main effect for time on skin temperature (*p* < 0.001, ηp2 = 0.92) was found**.** Mean skin temperature is shown in Figure 2B; where solid lines indicate N_2_O_2_ gas mix and dashed lines indicate HeO_2_. Mean skin increased from baseline (prior to donning wet-suit) to pre-splash (wet-suit donned), for all dive depths and gas mixes. As with core temperature, this is likely due to the increased metabolic heat production (from movement) during the time required to don wet-suit and equipment while dry. After descent to the predetermined depth, skin temperature (independent of dive depth or gas mix) rapidly declined and plateaued between 60–90 min when the skin reached a state of thermal equilibrium with the environment. Dives elicited a significant decrease in mean skin temperature (30fsw (9.1 m) pre: 33.1 ± 0.2, post: 25.8 ± 0.5; 50fsw (15.2 m) pre: 33.6 ± 0.3, post: 23.9 ± 0.4; 75fsw (22.9) pre: 32.8 ± 0.5, post: 23.6 ± 0.8; *p* < 0.001). The lowest individual mean skin temperature recorded was 20.0°C (68.1°F); which occurred following the 75fsw dive using HeO_2_. The respective adjusted R2 indicates that time accounts for 87%–94% of the variance with respect to rate of mean skin temperature change. When analysed by gas for each dive depth, the adjusted R2 values indicate that time accounts for 75%–96% of the variance when breathing N2O2 and 90%–93% of the variance when breathing HeO2.

Mean Hand and Finger Temperature. There were no significant main effects for depth or gas, but there were significant main effects for time on hand temperature (*p* < .001, ηp2 = .98). Mean hand temperatures, obtained from patch on skin, are depicted below in Figure 2C, where solid lines indicate breathing N_2_O_2_ gas mix and dashed lines indicate breathing HeO_2_. Mean hand temperatures were not measured for the 30fsw (9.1 m) dive breathing N_2_O_2_ (Dive 1) due to error. At dive onset, hand temperatures rapidly decline to a greater degree than core and mean skin temperatures due to the distal proximity to the heart. Regardless of dive depth and/or gas mix, mean hand temperatures decrease until approximate dive time of 90min of dive where thermal equilibrium occurred. Two dives were terminated due to divers’ hand temperatures decreasing below 10.0°C. Dive 2 (30fsw (9.1 m), N_2_O_2_) was terminated when one diver’s hand reached 9.9°C (49.8°F) at 60 min. The diver indicated that their gloves were not donned correctly, and the gloves were worn outside of the wet-suit when normally the gloves are placed under the wrist of the wet-suit. Dive 5 (50fsw (15.2 m), HeO_2_) was terminated, due to one diver’s hand reaching termination threshold of 10.4°C (50.7°F).

The respective adjusted R2 indicates that time accounts for 72%–91% of the variance with respect to the rate of mean hand temperature change. When analysed by gas for each dive depth (Figure 2B), the adjusted R2 values indicate that time accounts for 69%–76% of the variance when breathing N2O2 and 88%–94% of the variance when breathing HeO2. Finger temperatures significantly decreased across all dives (pre:25.4 ± 0.7, post: 17.4 ± 1.2; *p* < 0.001). Regression model indicates that 61% of finger temperature variance can be attributed to the temperature of the hand. There was a strong correlation between hand and finger temperature across all dives (*R* ^2^ = 0.61). Pre and post hand and finger temperatures are in Table 2.

**TABLE 2:** Extremity temperature by dive depth and gas. There were no significant main effects for depth or gas, but there were significant main effects for time on hand temperature (*p* < .001), finger (*p* < 0.001), foot temperature (*p* < 0.001) and toe (*p* < 0.001). There were also significant interaction effects in foot temperature for depth and time (*p* = 0.04) as well as depth, gas, and time (*p* = 0.004). * Indicates significant for time, #-significant for depth and gas.

	N2O2		HeO2	
	Pre	Post	Pre	Post
**Depth 30′**				
Hand (°C)			26.4 ± 1.8	20.10 ± 1.1*
Finger (°C)	23.9 ± 0.8	16.7 ± 1.6*	24.4 ± 0.7	15.80 ± 2.3*
Foot (°C)	29.4 ± 1.2	17.7 ± 1.8*	28.5 ± 0.6	14.24 ± 1.5*
Toe (°C)	24.4 ± 1.0	13.9 ± 1.4*	22.1 ± 0.4	9.93 ± 1.0*
**Depth 50′**				
Hand (°C)	28.4 ± 1.1	13.7 ± 1.4*	26.4 ± 1.8	14.4 ± 1.7*
Finger (°C)	23.9 ± 0.8	16.7 ± 1.6*	24.2 ± 0.7	12.4 ± 0.6*
Foot (°C)	29.4 ± 1.2	17.7 ± 1.8*	28.5 ± 0.6	13.8 ± 0.7*
Toe (°C)	24.4 ± 1.0	13.9 ± 1.4*	22.1 ± 0.4	11.7 ± 2.0*
**Depth 75′**				
Hand (°C)	28.7 ± 0.7	18.2 ± 2.0*	26.1 ± 2.0	18.3 ± 0.8*
Finger (°C)	27.6 ± 2.6	15 ± 1.3*	26.1 ± 2.0	20.2 ± 0.5*
Foot (°C)	27.5 ± 2.3	13.3 ± 1.7*#	31.7 ± 0.0	20.6 ± 0.3*#
Toe (°C)	21.8 ± 0.1	11.9 ± 1.7*	26.2 ± 2.0	16.5 ± 0.9*

Mean Foot and Toe Temperature. There were no significant main effects for depth or gas, but there were significant main effects for time on foot temperature (*p* < .001, ηp2 = .99). There were also significant interaction effects in foot temperature for depth and time, (*p* = .04, ηp2 = .61), as well as depth, gas, and time for 75fsw (*p* = .004, ηp2 = .84) Mean foot temperatures, obtained from patch on skin, are shown below in Figure 2D; where solid lines indicate breathing N_2_O_2_ gas mix and dashed lines indicate breathing HeO_2_. Pre and post foot and toe values are in Table 2. As observed with hand and finger temperatures, at dive onset, foot temperature rapidly declined and to a greater degree than mean core, skin, and hand temperatures. Foot temperatures continuously decreased until dive termination. The respective adjusted R2 indicates that time accounts for 69%–92% of the variance with respect to the rate of mean foot temperature change. When analysed by each gas for each dive depth (Figure 2D), the adjusted R2 values indicate that time accounts for 76%–94% of the variance when breathing N_2_O_2_ and 93%–97% of the variance when breathing HeO2. Toe temperature decreased significantly (pre: *p* < 0.001) and that there is a strong correlation between foot and toe temperature (R2 = 0.86) which indicates that variance in toe temperature can be attributed to the temperature of the foot.

## Discussion

The primary aim of this effort was to evaluate the effectiveness of a closed cell wet-suit on thermoprotection of military divers across three different depths and two gas mix. The main findings were that core temperature was maintained above the threshold of hypothermia across all depths and both gas mixture albeit it decreased significantly from dive onset. Mean skin temperature and extremity temperature decreased as a function of dive time. The change in digit (finger and toe) was largely driven by change in hand and foot temperature. The overall conclusion is that closed cell neoprene wet-suits are effective in preventing hypothermia and that extended time in cold-water (greater than 60 min) may increase risk of NFCI at extremities.

Core temperature maintenance is critical for vital organ function and for prevention of hypothermia. Throughout all dives, core temperature was preserved above the threshold for hypothermia, independent of dive depth and/or gas mix. There were slight variations in rate of core temperature decline between gas mix during the 50fsw dive. There was a larger rate of decrease during HeO_2_ than N_2_O_2_ during the 50fsw dive; however, it was not statistically different between the gas mix or between depths. Heat loss can occur from breathing both dry and cold compressed air as well as air mixed with various gases (Nakayama, 1978; Burnet et al., 1990; Passias et al., 1992). As the body warms and humidifies the air in the respiratory tract, there is both evaporative heat loss (humidifying) and convective heat loss (warming) (Piantadosi & Thalmann, 1980). However, in this effort closed circuit rebreather was utilized which recycles and clean the diver’s air. As such, the divers were breathing warm air and thus this was not a factor effecting core temperature in this effort. Previous work suggests that gas mix may modulate thermoregulatory response to cold water (Passias et al., 1992; Lun et al., 1993) but in this sample we did not observe a significant difference which concurs with others (Hoar et al., 1976; Spitler et al., 1980). This may be due the depth not being as deep or the duration of HeO_2_ exposure time as long compared to other efforts that found an effect on core temperature with HeO_2_ (Kuehn & Zumrick, 1978; Piantadosi & Thalmann, 1980). The participants in this effort were experienced divers; however, they routinely train in moderate temperature water (25.4°C) and thus were not habituated to the cold. Previously we demonstrated an increase in metabolic rate in an analogous population under similar conditions (Chapin et al., 2021). While not directly measured in this effort, it can be assumed that there was in increase in metabolic thermic response (Brazaitis et al., 2014). The participants in this effort were highly fit military divers and high rates of shivering were noticed anecdotally upon exit from the OSF. While not quantified, shivering thermogenesis is a well-known mechanism by which the human body produces heat to preserve core temperature (Haman, 2006). The diver’s core region had two layers of neoprene due to the style of wet-suit worn. While compression of the wet-suit is expected (Monji et al., 1989), it is likely that the additional thickness in the core region contributed to core temperature maintenance. Internal single cycle compression testing demonstrated the 10 mm wet-suit compressed to 4 mm at 75fsw which would provide approximately 8 mm of protection at the core. Thus, core temperature preservation above hypothermia could be attributed to a combination of wet-suit thickness at the core, body composition and fitness of the participants in this effort (Jacobs et al., 1984; Glickman-Weiss et al., 1991; Jensen et al., 2019).

Coupled to core temperature regulation is mean skin temperature. Vasoconstriction of the periphery is a normal physiological response to preserve core temperature. Cold skin stimulates an adrenergic response to shunt blood from the periphery to the thoracic cavity. However, it often comes at the expense of the extremities. Vasoconstriction and the high surface area-to-volume ratio results in a rapid and exponential decrease in skin temperature until ambient temperature is reached. Across all dives mean skin temperature significantly decreased rapidly within the first 30 min of the dive where it plateaued and stabilized. Regression analysis indicates that time accounts for 84%–94% of the variance across different dive depths. When gas was factored in, 75%–96% of the variance in skin temperature was attributed to breathing N_2_O_2_ and 90%–93% of the variance when breathing HeO_2_. While there are no cut-off points for skin temperature, skin temperature is critical in the regulation of blood flow to the extremities via modulation of blood flow to prevent heat loss (Zafren, 2021).

Mean hand temperature declined more rapidly and to a greater degree than mean core and skin temperatures (Table 2). Distal relationship to core increase risk of extremities for NFCI and can reduce dexterity (Havenith et al., 1995; Heus et al., 1995). Normally within 5–10 min of cold exposure, cold induced vasodilation occurs to send blood to the hands and fingers (Daanen et al., 1997; Daanen, 2003; Imray et al., 2011). Changes in blood flow to the hands is related to both mean skin temperature and the rate of heat loss (Daanen et al., 1997; Imray et al., 2011; Zafren, 2021). While these data were collected in individuals immersed or exposed in bare skin; the physiological response is the same for a thermoprotected individual as the response is dictated by skin temperature (Haman et al., 2022). In this effort, two dives were terminated due to hand temperatures reaching 10°C which was the threshold for termination criteria as there is an increased risk for NFCI. While the pathology behind NFCI is not well understood and interindividual responses vary (Tipton et al., 2017); it is know that NFCI can result in a lifetime of cold sensitivity and pain and is associated with military training (St Clair Golden et al., 2013; Sullivan-Kwantes & Tikuisis, 2023; Tipton & Eglin, 2023). Further, while clinical metrics for NFCI were not collected in this effort, observations of extremity pain, numbness and color change were noted. There is very limited data on the risk of NFCI in a diving population (Haman et al., 2022; Sullivan-Kwantes & Tikuisis, 2023) and thus future work should address not only changes in extremity temperature, but quantifiable metrics related to NFCI. While prevention of NFCI is critical, more importantly, it is well known that at hand temperatures of 20°C there begins a degradation in performance and dexterity (Havenith et al., 1995; Heus et al., 1995). In this effort hand temperatures were at this threshold or slightly below during every dive. With respect to occupational performance, this metric is critical to conduct occupational tasks, and life-saving rescue procedures. From these data, at depths greater than 30fsw, hand dexterity is compromised after 60 min of exposure. Thus, when considering the use of wet-suits, time in cold water is an important consideration.

Like hand temperature, foot temperature (Figure 2D; Table 2) decreased rapidly upon entry into the cold water and plateaued at 60 min for all three dives. There were no differences between gas mix except for the 75fsw dive, with N2O2 eliciting colder foot temperatures. While this is statistically significant, it may not be clinically relevant. More work is needed to determine if there is relevance to this finding. Traditionally, breathing HeO2 yield colder core temperatures; however, there are limited data on the effects on extremity temperature by which to reference. Nevertheless, across all dives, foot temperature continued to decline to approximately 14°C which is just above the threshold for a NFCI and potential neural damage (Tipton et al., 2017; Zafren, 2021). To reduced risk of potential injury, divers utilizing wetsuits should be aware of prolonged exposure. While the use of the lower the use of lower extremity has not been quantified to the extent of the hand, the ability to egress from the water and maintain mobility of the foot is important for military operations.

## Limitations

There are several limitations to address. Primarily, the sample is limited in that these data are from a small military population of highly trained divers with good fitness levels. Second, there are no females represented in this cohort. Compression testing of the wetsuits was conducted internally and only a single cycle of descent and ascent was done and so accurate deformation and elastic recoil property of the wet-suit are not quantified. Shivering onset time were not recorded and shiver rate was not measured. Further, the divers were motionless in this effort and thus changes in thermoprotection may alter with flushing of cold water into the wet-suit which can occur with movement.

## Conclusion

The primary findings of this effort are that closed-cell neoprene wet-suits are adequate for thermal protection against hypothermia and NFCI for use in cold-water dives with the understanding that depth and duration are critical considerations. Based upon the aggregated findings from these data 60 min is a safe time threshold to reduce risk of NFCI at the extremities. Moreover, in this effort, there was no effect of gas mix on thermoregulation across all depths except for at the foot during the 75fsw. The greatest occupational limitation that should be considered is change in dexterity especially as time in cold water persists. While not directly measured in this effort, previous data suggest that hand temperatures below 15°C decrease dexterity (Heus et al., 1995). Thus, it can be inferred that at temperatures below this the threshold ability to not only conduct skilled tasks but self-rescue in the event of an emergency may be compromised. Nevertheless, these data provide additional use case scenarios for the use of wet-suits as an alternate thermoprotective garment to dry-suits. Future work is warranted to evaluate the change in dexterity [hand] as well as mobility [foot] during cold-water diving as well as egress from the water if by land and to determine the impact of movement [active finning] on thermoprotection.

## Data Availability

The raw data supporting the conclusion of this article will be made available by the authors, without undue reservation.

## Disclaimer

I am a military service member or employee of the U.S. Government. This work was prepared as part of my official duties. Title 17, U.S.C. §105 provides that copyright protection under this title is not available for any work of the U.S. Government. Title 17, U.S.C. §101 defines a U.S. Government work as work prepared by a military service member or employee of the U.S. Government as part of that person’s official duties.

The views expressed in this article are those of the authors and do not necessarily reflect the official policy or position of the Department of the Navy, Department of Defense, nor the U.S. Government. The study protocol was approved by the Naval Health Research Center Institutional Review Board in compliance with all applicable Federal regulations governing the protection of human subjects. Research data were derived from an approved Naval Health Research Center Institutional Review Board protocol number NHRC. 2017.0019.
